# Supplementary figures and images for: LPCAT1 enhances castration resistant prostate cancer progression via increased mRNA synthesis and PAF production
Source: PLoS One. 2020 Nov 2;15(11):e0240801. doi: 10.1371/journal.pone.0240801 (PMC7605678; doi:10.1371/journal.pone.0240801)

Fig1

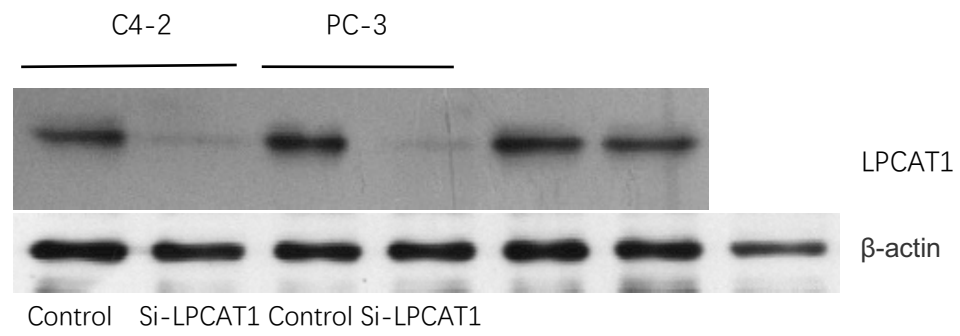

Fig2

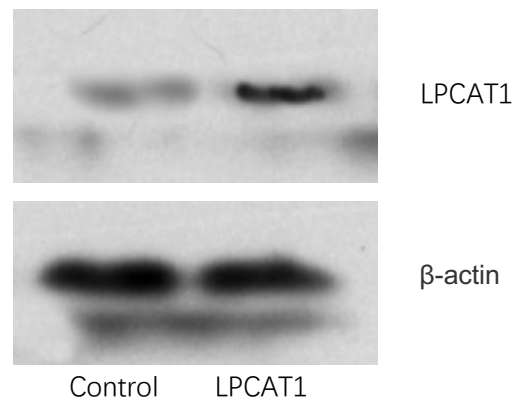

Fig 4

A

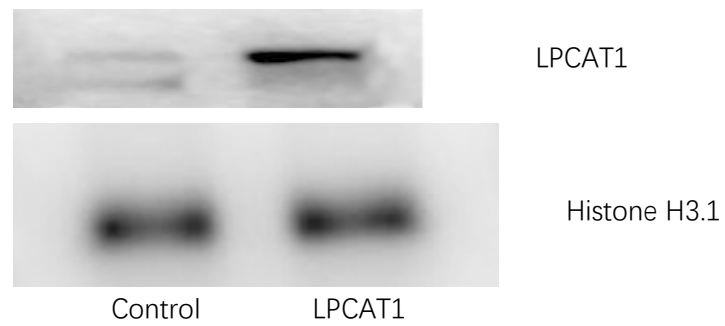

B

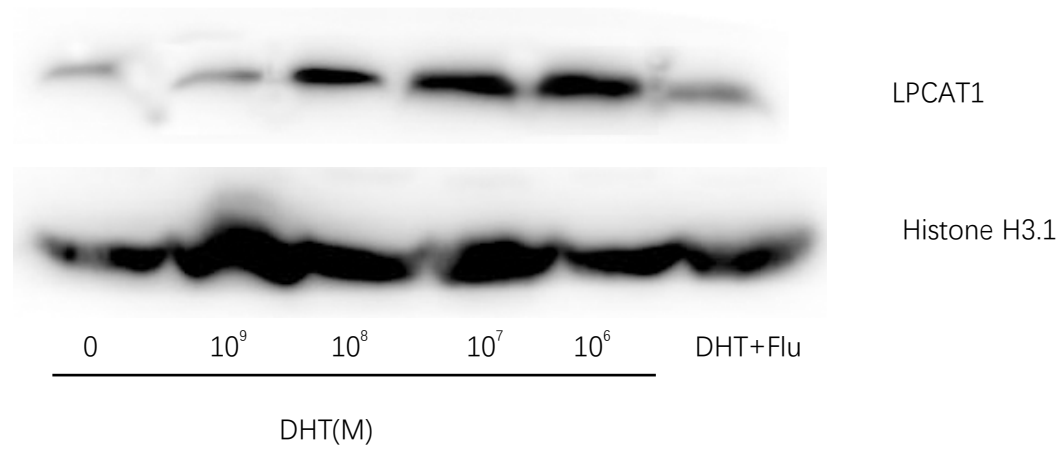

E

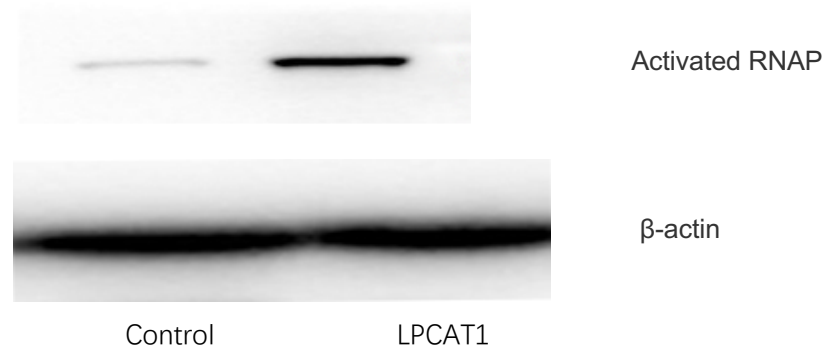

F

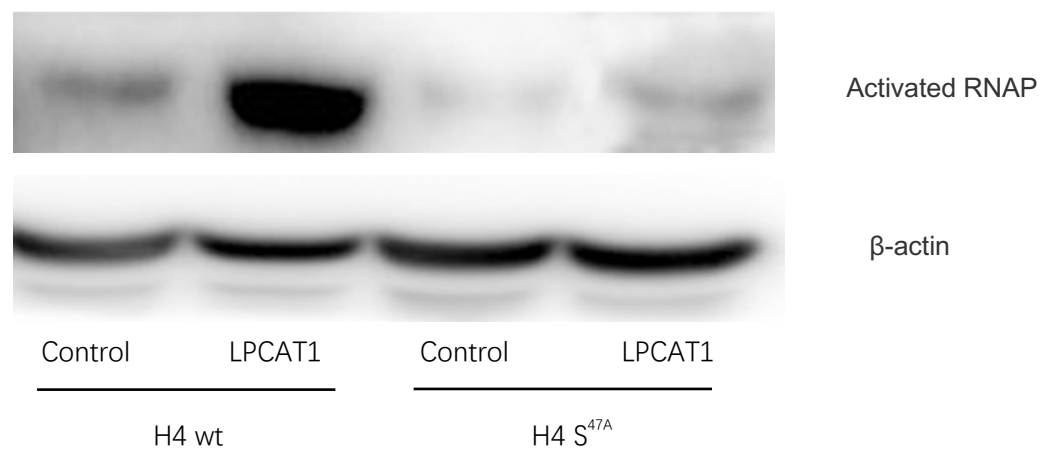

Fig 1A

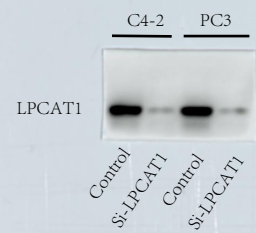

Fig 1A

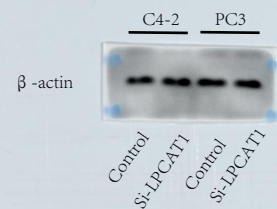

Fig 2A

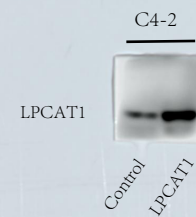

Fig 2A

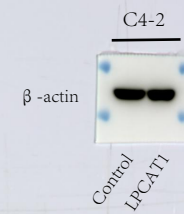

Fig 4A

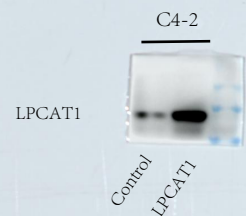

Fig 4A

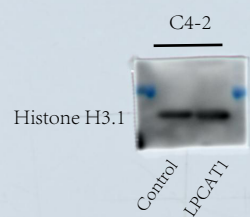

Fig 4B

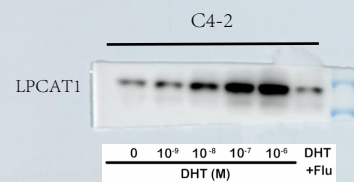

Fig 4B

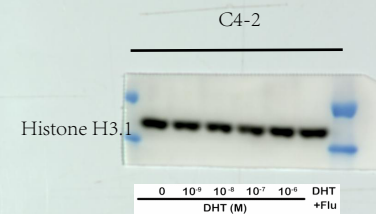

Fig 4E

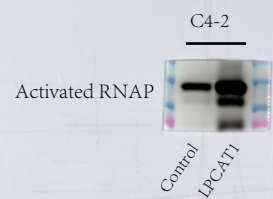

Fig 4E

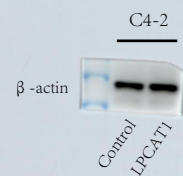

Fig 4F

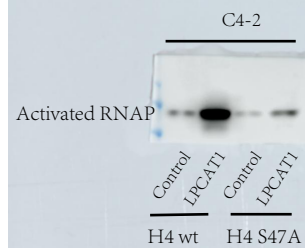

Fig 4F

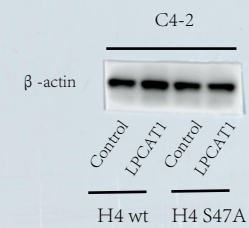

Supplement: S1 Original images — (PDF) [file pone.0240801.s001.pdf]
