# Supplementary material for: LPCAT1 enhances castration resistant prostate cancer progression via increased mRNA synthesis and PAF production
Source: PLoS One. 2020 Nov 2;15(11):e0240801. doi: 10.1371/journal.pone.0240801 (PMC7605678; doi:10.1371/journal.pone.0240801)
Supplement: S1 File — (DOCX) [file pone.0240801.s002.docx]

**1. Study design**

To further verify the phenotypes we observed in vitro，we did similar experiments in nude mice. we conducted three xenograft tumor experiments to assess (1) whether LPCAT1 overexpression can promote tumor growth, (2) how androgen influenced tumor growth in the presence or absence of LPCAT1 overexpression and (3) cell sensitivity to chemotherapeutic agents upon LPCAT1 overexpression. The specific experimental procedures are detailed below.

**2. Sample size**

Total eighteen mice were used and each xenograft tumor experiment contained six mice.

**3. Inclusion and exclusion criteria**

We selected 8-week-old male BALB/c nude mice, weighing 22–24g.

**4. Randomisation**

Eighteen mice were randomly divided into two groups (9 mice/group): the overexpressing group and the control group.

**5. Blinding**

We could not be blinded to the mice due to the different treatment.

**6. Outcome measures**

Mice were monitored every 3 days and were sacrificed before the tumor volume reached 1000 mm^3^. The tumor volume of all mice was less than 1000mm^3^ in 6 weeks after transfection and no mice died before meeting criteria for euthanasia. In order to alleviate suffering, all mice were sacrificed by cervical spine dislocation after anesthesia by Isoflurane.

**7. Statistical methods**

Results are given as means ± s.e.m. Differences between groups were assessed via one-way ANOVA and Student-Newman-Keuls test. The significance threshold was P<0.05.

**8. Experimental animals**

Eighteen 8-week-old male BALB/c nude mice, weighing 22–24g, were obtained from Slaccas Laboratory Inc.(Shanghai, China).

**9. Experimental procedures**

Eighteen 8-week-old male BALB/c nude mice, weighing 22–24g, were obtained from Slaccas Laboratory Inc.(Shanghai, China) and randomly divided into two groups (9mice/group): the overexpressing group and the control group. A plasmid encoding for LPCAT1 or empty vector control was transfected into C4-2 cells. The overexpressing group mice were injected LPCAT1-overexpressing C4-2 cells and the control group mice were injected empty vector control C4-2 cells. Following transfection, cells (overexpressing group and control) were suspended in a 50:50 mixture of PBS and Matrigel (BD, Franklin Lakes, NJ, USA), and 3 × 10^6^ cells were subcutaneously injected into the flanks of these mice. Mice were monitored every 3 days and were sacrificed before the tumor volume reached 1000 mm^3^. The tumor volume of all mice was less than 1000mm^3^ in 6 weeks after transfection and no mice died before meeting criteria for euthanasia. In order to alleviate suffering, all mice were sacrificed by cervical spine dislocation after anesthesia by Isoflurane.

To assess whether LPCAT1 overexpression can promote tumor growth, 6 mice (3 overexpressing and 3 control) were euthanized and tumors were harvested and weighed in 6 weeks after transfection.

To assess how androgen influenced tumor growth in the presence or absence of LPCAT1 overexpression, 6 mice (3 overexpressing and 3 control) ’s murine testes were removed 2 weeks following tumor cell implant. After 6 weeks, 6 mice were euthanized and tumors were harvested and weighed.

To assess cell sensitivity to chemotherapeutic agents upon LPCAT1 overexpression, paclitaxel (20mg/kg) or DMSO vehicle control was injected intra-peritoneally into 6 mice (3 overexpressing and 3 control)(i.p.) daily for 5 days, starting 14 days after tumor cell implant as previously described. After a total of 6 weeks, 6 mice were sacrificed as above, with tumor volumes being measured every two days.

**10. Results**

These experiments demonstrated increased tumor growth in cells expressed elevated levels of LPCAT1, there was no significant difference in tumor weight between LPCAT1 overexpressing group and control when male nude mice was castrated at 6 weeks and LPCAT1 renders CRPC cells resistant to paclitaxel therapy in vivo.
